# Minimal Influence of Cayenne Pepper on the Human Gastrointestinal Microbiota and Intestinal Inflammation in Healthy Adult Humans—A Pilot Study

**DOI:** 10.3390/life12111849

**Published:** 2022-11-11

**Authors:** Sihan Bu, Wreeti Kar, Robin M. Tucker, Sarah S. Comstock

**Affiliations:** Department of Food Science and Human Nutrition, Michigan State University, East Lansing, MI 48824, USA

**Keywords:** calprotectin, lipocalin-2, capsaicin, tomato juice, *Bifidobacterium*, *Gp6*, *Phascolarctobacterium*, *Oscillibacter*

## Abstract

Diet impacts human gut microbial composition. Phytochemicals in cayenne pepper (CP), such as capsaicin, have anti-inflammatory properties and alter bacterial growth in vitro. However, the evidence that CP impacts the human microbiota and intestinal inflammation in free-living adults is lacking. Thus, the objective of this randomized cross-over study was to determine the influence of CP on human gut microbiota and intestinal inflammation in vivo. A total of 29 participants were randomly allocated to consume two 250 mL servings of tomato juice plus 1.8 g of CP each day or juice only for 5 days before crossing over to the other study arm. Fecal samples were analyzed. CP reduced *Oscillibacter* and *Phascolarctobacterium* but enriched *Bifidobacterium* and *Gp6*. When stratified by BMI (body mass index), only the increase in *Gp6* was observed in all BMI groups during CP treatment. Stool concentrations of lipocalin-2 and calprotectin were similar regardless of CP treatment. However, lipocalin-2 and calprotectin levels were positively correlated in samples taken after CP consumption. Neither lipocalin-2 nor calprotectin levels were related to gut microbial composition. In conclusion, in healthy adult humans under typical living conditions, consumption of CP minimally influenced the gut microbiota and had little impact on intestinal inflammation.

## 1. Introduction

The human gastrointestinal tract is a complex ecosystem containing abundant microorganisms that play specific functions in host nutrient metabolism, the regulation of intestinal inflammation and the host immune system, and maintenance of gut barrier integrity [1,2,3]. The two predominant phyla of a healthy gut microbiota are *Firmicutes* and *Bacteroidetes* followed by *Actinobacteria*, *Proteobacteria*, *Fusobacteria*, and *Verrucomicrobia*, [4]. The phylum *Firmicutes* consists of more than 200 genera, such as *Phascolarctobacterium*, *Oscillibacter, Lactobacillus* and *Ruminococcus* [5]. The phylum *Actinobacteria* is mainly represented by *Bifidobacterium* [6].

Most evidence available today demonstrates that diet is an essential factor impacting the composition of the human gut microbiota as gut microbial composition can be modified by short-term and long-term dietary alterations [7,8,9,10,11]. One dietary component that deserves further study related to its impact on the gut microbiome is spice. Spices, such as black pepper, cayenne pepper, cinnamon, ginger, and turmeric, contain phytochemicals, including polyphenols and phenolic acids [12]. Dietary polyphenols have antioxidant, anti-inflammatory, anti-cancer, and anti-diabetic properties, indicating a relationship between human health and polyphenol-rich food consumption [13]. Many ingested phytochemicals remain in the intestine because of poor absorption, and these molecules are metabolized by gut microbes [14,15]. With regards to bacteria, some polyphenols can function as prebiotics and promote the growth of *Bifidobacterium* spp. and *Lactobacillus* spp. as well as inhibit the growth of *Ruminococcus* spp. [12]. However, little research has addressed the potential link between dietary intake of spices and changes in the human gut microbiota.

The relationship between spices and intestinal inflammation has steadily gained more consideration in recent years. Intestinal inflammation is the response that occurs within the mucosal barrier from the invasion of a microbial antigen and the ensuing immune response to that antigen [16,17]. Chronic intestinal inflammation can result in permanent mucosal barrier damage and bowel injury [18]. There is mounting evidence indicating that certain spices can aid digestive activity and have anti-inflammatory effects [19]. For example, active chemical compounds in cinnamon (*Cinnamomum verum*) have anti-microbial properties [20]. Curcumin, a molecule found in turmeric, inhibits T cell-mediated immune functions, such as cellular proliferation, and plays a crucial role in chronic intestinal inflammatory disease [21,22]. Capsaicin, an important phenolic compound present in chili peppers, inhibits the production of pro-inflammatory mediators such as IL-1β, IL-6, and TNF-α in vitro [23,24]. Additionally, spices appear to reduce the inflammation in the context of human gut microbiota. Cayenne pepper contains high amounts of capsaicin, and its administration in cell culture exhibited a prebiotic effect—increasing beneficial bacteria while suppressing pathogenic bacteria [12]. These effects may contribute to reduced numbers of pathogenic bacteria available to adhere to colonocytes, which might reduce the intestinal inflammatory response [25]. Thus, spices are likely an important dietary component for reducing intestinal inflammation.

There has been limited research to determine if cayenne pepper (CP) influences the human gut microbiota diversity and intestinal inflammation in vivo. Prior intervention with a 1 g dose of CP did not have pronounced effects on the gut microbiota of free-living human study participants [26]. Thus, the objective of this single-crossover dietary intervention study was to investigate whether a higher amount (1.8 g) of CP alters the gut microbial composition and affects fecal biomarkers of intestinal inflammation in vivo. We hypothesized that CP would enrich *Bifidobacterium* but reduce *Ruminococcus* in the gastrointestinal microbiomes of adults.

## 2. Materials and Methods

### 2.1. Study Population

In this cross-over pilot study, a total of 44 consenting participants were recruited by convenience sampling through the Michigan State University Sona Research Participant system between March 2019 and January 2020. The participants were recruited if they were healthy nonsmokers, between 19 and 55 years old, and were not habitual consumers of spicy foods [27]. Participants who were at risk of gut microbiota dysbiosis or tomato allergies were excluded from this study. We also excluded individuals taking nutritional supplements, nonsteroidal anti-inflammatory medications, and antibiotics 2 weeks before or during the study. All participants gave written, informed consent. Height and weight were measured at the first study visit. We classified the participants into 2 groups based on their BMI (BMI = body weight in kg divided by height in meters squared): underweight and normal weight (BMI < 25) and overweight and obese (BMI ≥ 25).

### 2.2. Intervention

**Figure 1:**
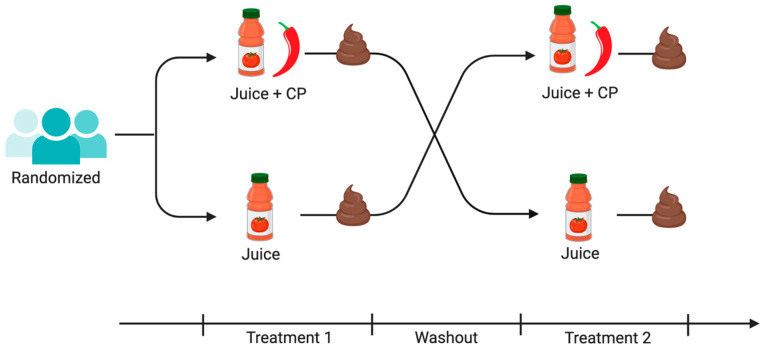
The study was a randomized cross-over intervention study. Each treatment period was five days. The washout period was two days. Figure made at biorender.com.

During the study, participants consumed (1) two 250 mL servings of tomato juice or (2) two 250 mL servings of tomato juice, where each serving contained 0.9 g of CP, each day for 5 days, and then observed a two-day washout period before crossing over to the other study arm (Figure 1). Tomato juice was chosen because in previous work it was shown that spicy tomato juice (1 g of red chili powder per 200 mL serving of tomato juice, equal to 2.0 mg capsaicin) was hedonically acceptable [28]. Herein, the CP consumption for an average participant was 0.021 g per kg of body weight. This dose is based on participants in this study, where the average body weight was 85 kg. Considering that 1 g of CP has about 2.5 mg of capsaicin [29], the participants had approximately 0.053 mg of capsaicin per kg body weight daily. The study design allowed participants to continue eating a mixed, complex diet, but sources of capsaicin, a component of CP, were to be avoided. G*Power was used to calculate the sample size setting alpha at 0.05, power at 0.80, and using a medium effect size (d = 0.5). A total of 34 participants must complete testing to achieve 80% power. An attrition rate of 25% was estimated based on prior studies in the laboratory of the investigator [28], so of the goal was to recruit at least 43 participants.

### 2.3. Sample Collection

**Figure 2:**
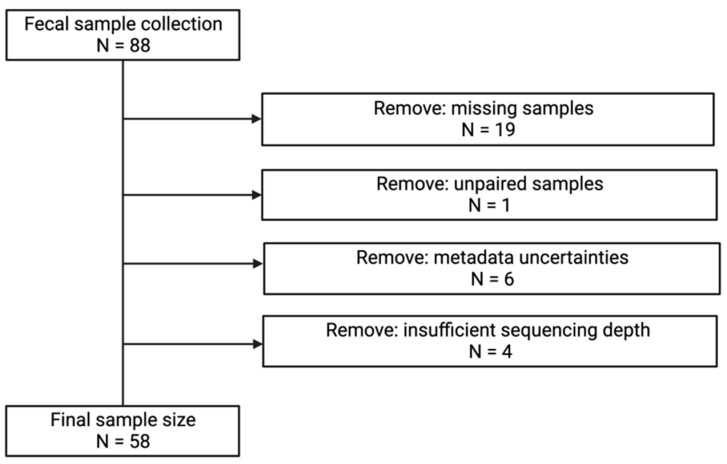
Sample flow chart.

Stool samples were collected at the end of each treatment and kept at room temperature in the participant’s home until brought to the lab within 24 h of collection. Stool samples were aliquoted, and then stored in a −80 °C freezer. After removing samples from our analyses due to either missing stool samples (19) or non-submittance of two stool samples (1), metadata uncertainties (6), or insufficient sequencing depth (4), the final sample size was 58 samples (Figure 2). This study was reviewed and approved by the Michigan State University Biomedical and Health IRB (IRB: 00001062) and aligned with the Declaration of Helsinki of 1975.

### 2.4. Laboratory Procedures

#### 2.4.1. DNA Extraction and 16S rRNA Gene Amplification

DNA extraction, 16S rRNA gene amplification, and sequencing were carried out on stool samples as previously described [30]. We used the Qiagen Powersoil DNA Isolation kit (Qiagen, Carlsbad, CA, USA) for DNA extraction; 16S V4 primers were purchased from Integrated DNA Technologies (Coralville, IW, USA) and were as described in the Schloss lab’s mothur wet lab standard operating procedure (SOP) (SB501-SB508 and SA701-SA712) [31]; AccuPrime Pfx SuperMix (ThermoFisher Scientific, MA, USA) as the DNA polymerase; Agencourt AMPure XP for PCR purification (Beckman Coulter, Brea, CA, USA) and sequenced at the Michigan State University Research Technology Support Genomics Facility using an Illumina MiSeq and 250 bp paired-end sequencing with v2 chemistry.

#### 2.4.2. Processing and Analysis of Sequence Data

Sequences were analyzed in mothur using the Illumina MiSeq SOP [32]. Sequence reads were binned according to the opticlust algorithm using a cutoff of 0.03 [33]. Taxonomic and phylogenetic data were generated using the SILVA reference database (release 102). The average number of reads per sample included in the final analysis was 145,777. Sample reads were rarefied to 15,000 reads per sample before further analysis, and adequate microbial community coverage was confirmed by rarefaction curves. For these analyses, the focus was on bacterial genera.

#### 2.4.3. Protein Extraction from Stool Samples

Extraction buffers for lipocalin-2 (Cat. No.30757) and calprotectin (Cat. No. 30473) were purchased from Epitope Diagnostics, Inc. (San Diego, CA, USA). Then, approximately 100 mg of fecal material was aliquoted into a conical tube. The exact weight of the stool sample was determined by subtracting the weight of the empty tube from the weight of the tube with stool. Using a serological pipette, 4 mL of extraction buffer per 100 mg stool was aliquoted into a conical tube for each sample. Each sample was extracted with each extraction buffer separately. Each tube was vortexed gently and incubated at room temperature on an orbital shaker for 30 min. After centrifugation, the protein extracts were aliquoted into microfuge tubes and stored in a −80 °C freezer.

#### 2.4.4. Enzyme-Linked Immunosorbent Assays (ELISAs)

Kits were purchased from R&D Systems (Minneapolis, MN, USA) for human calprotectin (S100A8/S100A9; DS8900) and human lipocalin-2 (NGAL; DLCN20). Fecal extracts were diluted 1:25 or 1:100 in sample diluent for calprotectin assays and 1:20 or 1:100 in sample diluent for lipocalin-2 assays. Assays were run as described in the product information manual. A PerkinElmer multimode plate reader from PerkinElmer, Inc. (Waltham, MA, USA) was used. After dilution, all sample protein concentrations fell within the standard curves of the assays. Final protein concentrations were determined using the standard curves and accounting for sample dilution. They are reported as ng stool per mL extraction buffer.

### 2.5. Statistical Analyses

Participant characteristics were expressed as mean ± standard deviation (SD). Microbiome data were analyzed using R (version 4.0.2) (Auckland, New Zealand). Data normality was tested using Shapiro–Wilk. Alpha diversity (Chao1, inverse Simpson and Shannon indices) was calculated using the vegan package [34]. Non-parametric data were analyzed using a Wilcoxon signed-rank test. Normally distributed data were analyzed using paired *t*-test to compare between study arms. For beta diversity, Sorensen (community membership) and Bray–Curtis (community composition) dissimilarities were calculated using the vegan package and visualized by principal coordinate analysis (PCoA). Permutational multivariate analysis of variance (PERMANOVA) was performed using the adonis function in the vegan package to test for significant differences in beta diversity. Gut microbiota with similar microbiota community shifts upon consumption of CP were clustered using cosine similarities between vectors from the PCoA based on the Bray–Curtis dissimilarity matrix.

Negative binomial mixed models or zero-inflated negative binomial mixed models in the NBZIMM package were used to test differences in bacterial relative abundance between groups [35]. The top 18 most abundant taxa were analyzed. False discovery rate (FDR) correction with Benjamini-Hochberg method was used for multiple comparisons. Inflammatory protein concentrations were compared using proc corr for correlation test and proc GLM for paired *t*-test in SAS version 9.4 (Cary, NC, USA). Correlations of gut microbiota taxa at the genus level and lipocalin-2 and calprotectin levels (continuous variables) were determined using MaAslin. *p*-value < 0.05 considered significant. *q*-value < 0.1 considered significant. 

## 3. Results

### 3.1. Participant Characteristics

Participants (*n* = 29) submitting two stool samples had a mean age of 29.5 ± 9.9 (mean ± SD) years. On average, participant BMI was 29.1 ± 8.2 kg/m^2^ (mean ± SD). Most participants were white (41.4%) or Asian (37.9%). Furthermore, the majority of participants (69.0%) were female (Table 1).

### 3.2. Impact of CP Treatment on Gut Microbiota Diversity and Composition

**Figure 3:**
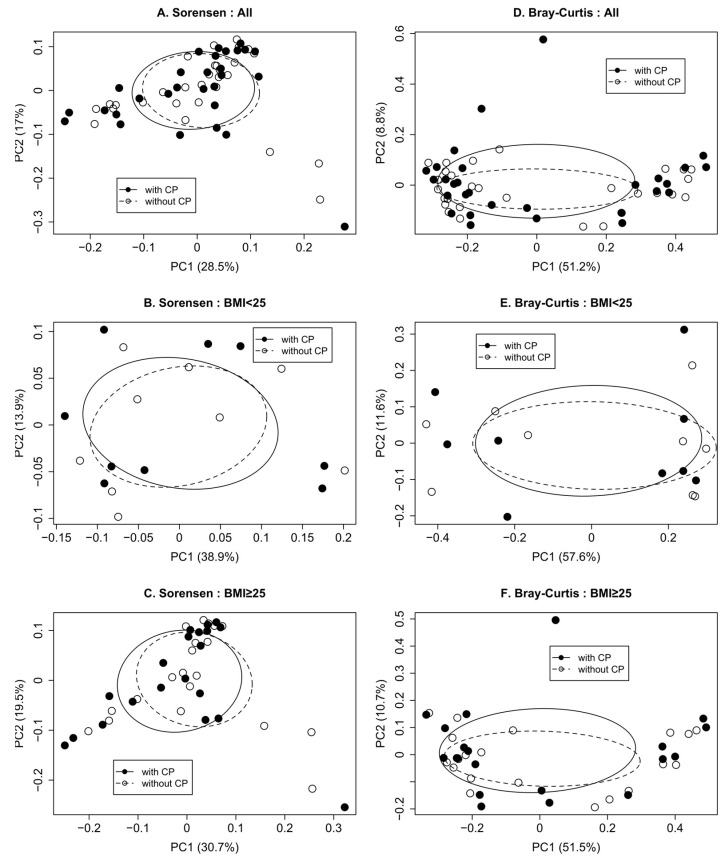
Whether consuming CP or not, gastrointestinal bacterial communities were similar. Beta diversity for all participants (**A**,**D**), those with BMI < 25 (**B**,**E**), and those with BMI ≥ 25 (**C**,**F**) were presented for membership (**A**–**C**) and community composition (**D**–**F**). *p*-values were (**A**) Sorensen, *p*-value = 0.99; (**B**) Sorensen, *p*-value = 0.93; (**C**) Sorensen, *p*-value = 0.99; (**D**) Bray-Curtis, *p*-value = 0.99; (**E**) Bray-Curtis, *p*-value = 0.99; (**F**) Bray-Curtis, *p*-value = 0.98. *p*-value < 0.05 was significant. BMI: body mass index.

CP treatment did not impact the microbiota alpha diversity. When stratifying by BMI, bacterial alpha diversity was similar in stool samples collected from the participants after the CP exposure and in stool samples collected when no CP was administered (Table 2). In the univariate analyses, CP was not associated with gut microbiota membership (Sorensen, Figure 3A) nor the gut microbiota composition (Bray–Curtis, Figure 3D) as determined at genus level without stratification. This was also true when stratifying by BMI (Figure 3B,C,E,F).

**Figure 4:**
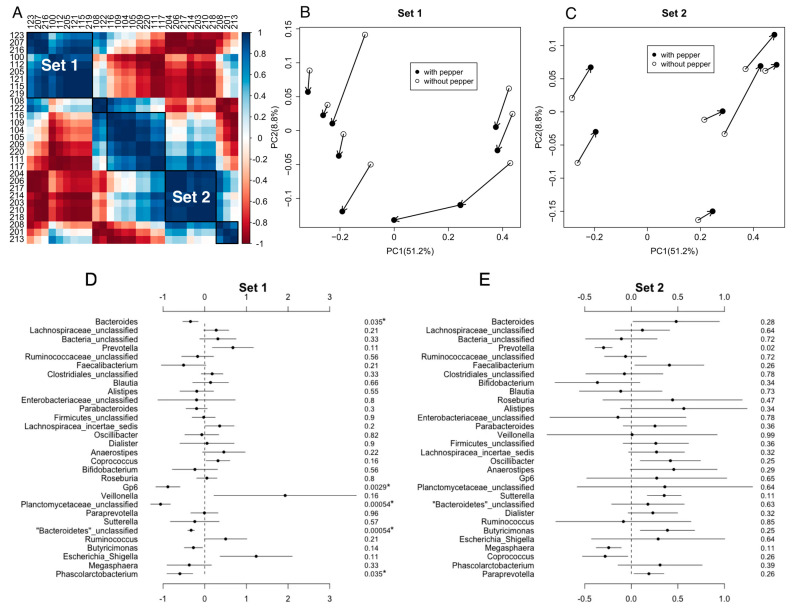
Beta Diversity of gastrointestinal bacterial communities by clustering the gut microbiota with similar microbiota community shifts upon consumption of CP. (**A**) Some participants (*n* = 16) could be binned into one of two groups-based cosine similarities. The remaining participants could not be binned based on cosine similarities. The axes are participant ID. The color gradients represent the cosine similarity. The dark blue is 1, and the dark red is −1. Principle coordinates analysis plots based on Bray–Curtis dissimilarities are shown in (**B**) Set 1 (*n* = 9) and (**C**) Set 2 (*n* = 7). Black dots represent each participant’s microbiota after consuming CP, whereas open dots represent each participant’s microbiota when not consuming CP. Comparisons of the abundance of the 30 most abundant taxa between CP exposure and non-exposure are shown in (**D**) Set 1 and (**E**) Set 2. The solid circle is the estimate of the coefficient, and the line indicates the confidence interval. The number in the right-most column is the FDR adjusted *p*-value for the statistical comparison. *p*-values with asterisks are significant, where *p*-value < 0.05 considered significant. The estimates are reported as the taxa abundance within the “without CP” group minus the taxa abundance in the “with CP” group. If the estimate is negative, the “with CP” group had a higher abundance. If the estimate is positive, the “without CP” group had a higher abundance.

Bar charts of the distribution of bacterial taxa at the phyla and genera levels are provided in Figure S1. Sixteen participants could be grouped into two sets based on global changes in their gut microbiota during the CP consumption arm of the study. Within each set, the participants had similar bacterial community shifts after consuming CP (Figure 4A–C). In Set 1 (*n* = 9), participants who consumed CP had a significantly higher abundance of *Bacteroides*, *Gp6*, unclassified *Planctomycetaceae*, unclassified *Bacteroidetes*, and *Phascolarctobacterium* than they did before consuming CP (Figure 4D). However, for participants in Set 2 (*n* = 7), only *Prevotella* was higher in those who were consuming CP (Figure 4E). Gut bacterial richness and evenness in Set 1 and Set 2 participants was similar regardless of CP consumption (Table S1). Notably, BMI did not differ between the groups (*p*-value = 0.15). Based on data collected during the first study visit, the mean and median BMI of Set 1 were 30.4 kg/m^2^ and 26.1 kg/m^2^, respectively. The mean and median BMI of Set 2 were 24.9 kg/m^2^ and 24.4 kg/m^2^, respectively. The remaining participants (*n* = 13) could not be grouped by overall changes in the gut microbiota composition.

**Figure 5:**
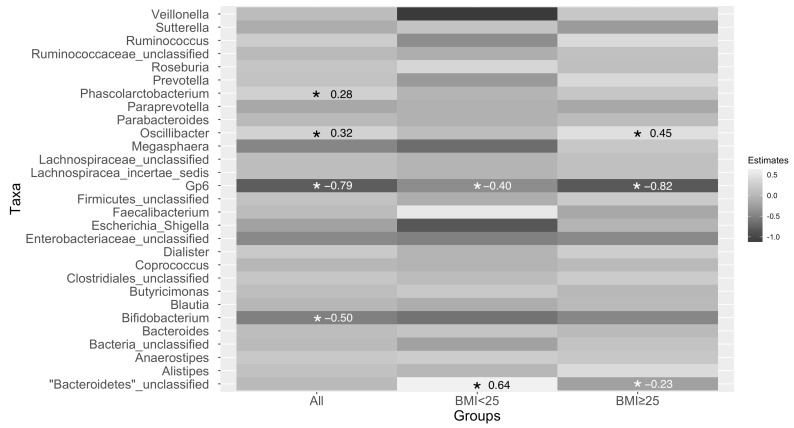
Changes in taxa abundance after 5 days of consuming CP in all participants combined (for left column) and when stratifying by BMI (middle and right columns). *p*-values were adjusted for multiple comparisons, and boxes with an “*” indicated that the adjusted *p*-value was <0.1 and significant. Estimates for the significant associations appear in each box with an asterisk. The estimates are reported as the taxa abundance within the CP group subtracted from the taxa abundance in the without CP group. If the estimate is negative, the with-CP group had a higher abundance. If the estimate is positive, the without-CP group had a higher abundance. BMI: body mass index.

The gut microbiota of participants had a significantly lower relative abundance of *Oscillibacter* and *Phascolarctobacterium*, but a higher abundance of *Bifidobacterium* and *Gp6* after consuming CP (Figure 5). When stratifying by BMI, the only consistent change in bacterial taxa was an increase in *Gp6*, whose relationship with human health is poorly understood. Gut microbiota of participants who had BMI < 25 had a significantly increased abundance of *Gp6* but a decreased abundance of unclassified *Bacteroidetes* after consuming CP (Figure 5). Participants who had a BMI ≥ 25 had a significantly lower abundance of *Oscillibacter* but a significantly higher abundance of unclassified *Bacteroidetes* and *Gp6* after consuming CP (Figure 5).

### 3.3. Effects of CP Treatment on Gut Inflammatory Biomarkers

**Figure 6:**
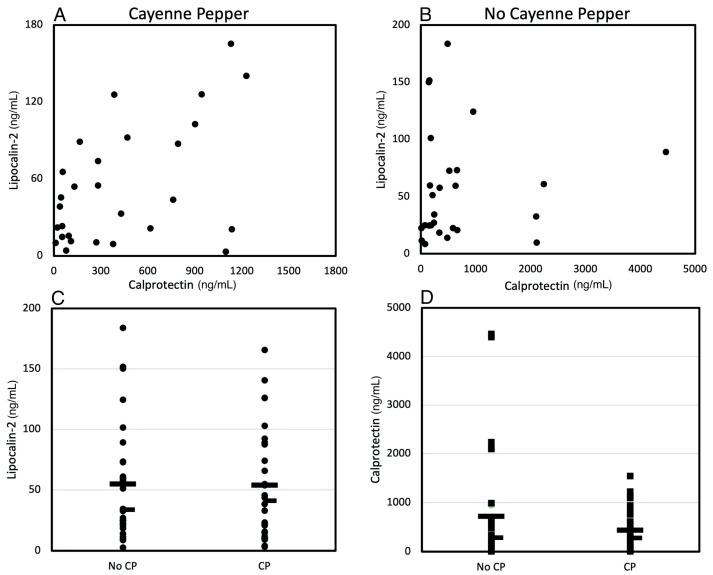
Stool biomarkers of inflammation. Calprotectin and lipocalin concentrations were correlated (**A**) (R^2^ = 0.4, *p*-value = 0.03) when participants were consuming tomato juice with CP but not when participants were consuming only tomato juice (**B**) (R^2^= 0.32, *p*-value = 0.32). Lipocalin (**C**) (*p*-value = 0.85) and calprotectin (**D**) (*p*-value = 0.56) concentrations were similar in stool samples from participants consuming tomato juice without or with CP. In panels **C**,**D**, the wide bar indicates the mean, and the narrow dash indicates the median. *p*-value < 0.05 considered significant.

Stool concentrations of lipocalin-2 and calprotectin were positively correlated in participants when consuming tomato juice with CP (Figure 6A, R^2^ = 0.4, *p*-value = 0.03) whereas no correlation was observed when participants were consuming tomato juice with no CP added (Figure 6B, R^2^ = 0.32, *p*-value = 0.32). However, the concentrations of either lipocalin-2 or calprotectin in stool samples collected from participants consuming tomato juice with CP were similar to levels in stool samples from participants consuming plain tomato juice (Figure 6C,D).

### 3.4. The Relationship between Gut Inflammatory Biomarkers and Gut Microbiota under CP Treatment

Neither lipocalin-2 (Figure S2) nor calprotectin (Figure S3) measures from the CP intervention group were related to gut microbiota membership (Figures S2B and S3B) or composition (Figures S2D and S3D). Similarly, neither lipocalin-2 nor calprotectin concentrations in the stools of participants who consumed only tomato juice were associated with the gut microbiota membership (Figures S2A and S3A) or composition (Figure S2C and S3C) using Sorensen and Bray–Curtis dissimilarities, respectively. Those with higher lipocalin-2 levels exhibited higher relative abundance of *Blautia*, *Anaerostipes*, *Alphaproteobacteria*_unclassified, and *Anaerosporobacter*. As calprotectin levels increased so did the relative abundance of *Eubacterium* and *Pseudoflavonifractor* (Table 3).

## 4. Discussion

Our study aimed to determine the influence of CP on human gut microbiota and intestinal inflammation in vivo. We found that CP consumption did not influence the gut microbial alpha and beta diversity with or without BMI stratification. Participants who consumed CP had a significantly lower relative abundance of *Oscillibacter* and *Phascolarctobacterium*, but a higher abundance of *Bifidobacterium* and *Gp6*. Stool concentrations of lipocalin-2 and calprotectin were similar before and after CP consumption. However, lipocalin-2 and calprotectin levels were positively correlated in the CP consumption group. Neither lipocalin-2 nor calprotectin levels were related to gut microbial composition. Thus, CP consumption at 1.8 g per day had minimal impact on either the gut microbiota composition or intestinal inflammation in this pilot study.

Emerging evidence suggests that polyphenol-rich foods, including spices, have potential as prebiotics that can promote the growth of probiotics, such as *Bifidobacterium* spp. and *Lactobacillus* spp., and inhibit the growth of pathogenic bacteria such as *Clostridium* spp. [12,36]. Culinary spices including black pepper, cayenne pepper, and cinnamon enhanced the growth of *Bifidobacterium* spp. And *Lactobacillus* spp. In vitro [12]. An increased abundance of *Bifidobacterium* and *Lactobacillus*, and a reduced abundance of *Clostridium* were found in participants who consumed a spice mixture containing cinnamon, oregano, ginger, black pepper, and cayenne pepper [36]. In addition to spices, red wine, wild blueberries and tart cherries also have been demonstrated to increase Bifidobacterial populations [37,38,39]. Numerous studies demonstrate that specific species of *Bifidobacteria* exhibit anti-infection properties, alleviation of lactose intolerance, and relief from constipation [40,41,42,43]. Therefore, our results that CP increased the abundance of *Bifidobacterium* contributes additional evidence that polyphenol-rich spices may have bifidogenic prebiotic properties. 

In a human trial, Khine et al. demonstrated that *Bacteroides* decreased with mixed spice intake among lean participants (average BMI = 22.9 kg/m^2^) [44]. In overweight participants (average BMI = 28.2 kg/m^2^), spice consumption resulted in a significant enrichment in *Bacteroidetes* [36]. Within our study, we noted a significant increase of unclassified *Bacteroidetes* among participants with BMI ≥ 25 but a decrease in this taxon among participants with BMI < 25 after consuming CP. Therefore, our results, along with previously published associations, indicate that the prebiotic effects of spices may be specific to host BMI.

The fact that our participants consumed tomato juice as the vehicle in this study may have confounded the impact of CP on the gut microbiota. Tomato juice consumption has been demonstrated to improve metabolic outcomes through changes to the gut microbiota. For instance, when rats were fed a high-fat diet, an increase in the abundance of *Lactobacillus* protected the rats from metabolic syndrome [45]. However, capsaicin has been demonstrated to reduce the abundance of *Lactobacillus* in the gastrointestinal microbiota of mice [46]. In other rodent research, mice fed a high-fat diet supplemented with capsaicin experienced an increase in fecal acetate concentrations compared to mice fed the high-fat diet exclusively [47]. On the contrary, consuming tomato juice decreased intestinal acetate among high-fat diet-fed rats [45]. Thus, the tomato juice and CP may have opposing effects in vivo. In the current study, we did not collect a baseline, “no tomato juice”, sample. However, it is worth noting that the effects of capsaicin in CP might have been offset by the use of tomato juice as the vehicle in this study. Thus, our use of tomato juice as a vehicle for the CP may have biased our results towards the null.

In our study, only changes in *Gp6* were consistent across BMI. In all instances, *Gp6* increased with CP exposure. Most bacterial changes upon consumption of CP were not consistent after stratifying by BMI. Indeed, populations of an unclassified *Bacteroidetes* shifted in opposite directions depending on BMI category. These discrepancies may be directly related to baseline differences in gut microbiota communities related to BMI or due to the small sample size in the underweight and normal weight (*n* = 9, BMI < 25) category when compared to the sample size for the overweight and obese (*n* = 20, BMI ≥ 25) category.

To determine the extent of intestinal inflammation, biomarkers such as calprotectin and lipocalin-2 were measured in fecal samples [48]. Calprotectin is released from neutrophils during inflammatory responses, is resistant to degradation, and is found in the stool making the measurements of calprotectin within the feces both practical and accurate [49,50]. During inflammatory bowel disease, high mucosal and fecal concentrations of lipocalin-2 occur [51,52,53], but lipocalin-2 can also be used as a more general biomarker of intestinal inflammation [54]. Previous studies in patients with inflammatory bowel disease observed a positive correlation between lipocalin-2 and calprotectin stool concentrations [55,56]. We also observed this positive correlation between lipocalin-2 and calprotectin in our study, but only when participants were consuming CP, indicating that the presence of CP in the diet may synchronize the release of these inflammatory mediators. An alternative interpretation is that CP decreased inflammation only in those individuals with the highest stool calprotectin levels. The five participants with stool calprotectin >2000 ng/mL did demonstrate a numeric decrease in calprotectin with CP treatment. However, the four individuals with stool calprotectin >1000 ng/mL but <2000 ng/mL had lower calprotectin concentrations when consuming plain tomato juice. Thus, this relationship requires further study. 

Lipocalin-2 is critically involved in maintaining intestinal microbiota homeostasis in mice [57]. In our study, we found that increased lipocalin-2 was correlated with increased relative abundance of *Blautia*. However, this is opposed to some studies which reported that the abundance of *Blautia* was significantly reduced in inflammatory bowel disease (IBD) patients compared to healthy individuals [58,59]. This might be due to differences in the severity of the gastrointestinal inflammation. The highest level of lipocalin measured in our study was 7.36 μg/g. Other studies have reported stool lipocalin level of 2.5 μg/g for healthy adult humans [60]. The relationship between calprotectin and gut microbiota has been also investigated among patients with intestinal disease. Children who had Crohn’s disease with calprotectin level <100 μg/g had higher richness and diversity of gut microbiota than those children with calprotectin levels between 100 μg/g and 1800 μg/g or above 1800 μg/g [61]. Ankylosing spondylitis (AS) is highly related to inflammatory bowel disease. AS patients with increased fecal calprotectin level (≥200 mg/kg) had lower abundance of gut bacteria with anti-inflammatory properties such as *Faecalibacterium prausnitzii* and *Clostridium* and higher abundance of the genus *Streptococcus* compared to patients with normal fecal calprotectin levels (≤50 mg/kg) [62]. The highest calprotectin level measured in our study was 1.55 μg/mL, where there were 25 mg of stool per ml of extraction buffer, and therefore this is equivalent to 61.9 μg of calprotectin per gram of stool after CP treatment, which is similar to the upper limit of normal, 50 μg/g [18] and much lower than levels observed in individuals with gastrointestinal inflammatory disease, 250 μg/g [63,64]. Thus, studies recruiting individuals with higher levels of intestinal inflammation may be more likely to observe an impact of CP treatment on the gut microbiota and/or intestinal inflammation.

Other limitations of this study design include a modest sample size and a failure to account for the time of day of stool sample collection as well as timing of sample collection with respect to most recent meal consumption. An additional limitation was the lack of a tomato-juice-only control. Tomato juice and CP may have opposite impacts as described above. Thus, a baseline sample of only tomato juice needs to be collected to control for this bias. Alternatively, future studies could employ a different vehicle for the CP. Both meal frequency and meal timing may impact normal peripheral and central circadian clocks, which in turn may affect the gut microbiota [65,66]. Moreover, the two-day washout period may be too short to eliminate the effects of cayenne pepper and for gut microbiota to return to a baseline state after a perturbation. A one-week washout period might be suggested. The unequal number of male (9) and female (20) participants is another limitation. Some studies suggest that adult males and females have distinct gut microbiotas, and these differences may be influenced by obesity [67]. Additionally, we did not measure the phytochemical composition in the commercial tomato and cayenne pepper products, which makes it difficult to know the exact amount of phytochemical exposure. Thus, the results of our intervention study cannot provide a level of phytochemical exposure that can impact the gut microbiota and inflammatory biomarkers. There might be the likelihood that the level of inflammation of the participants with BMI >25 is higher than those with BMI <25. Therefore, serum biomarkers can be used in future studies to determine more accurate inflammatory levels in participants rather than relying on BMI as a proxy for systemic chronic inflammation. Another limitation is the sample collection method. An ideal fecal collection method would include immediate processing or freezing at −80 °C [68]. However, in our study, all the fecal samples were collected at home and brought to the lab at room temperature. This could have impacted the bacterial abundances. However, all samples were collected and treated using the same procedure. Thus, there is within study reliability. However, caution should be taken when comparing these results with those reported elsewhere when samples are collected and processed differently than described herein.

## 5. Conclusions

Cayenne pepper intake of 1.8 g/day for 5 days minimally altered the overall composition of human gut microbial communities. This was true in all participants, regardless of BMI. Furthermore, the average BMI was similar in the two sets of individuals where consistent shifts in the overall gut microbiota composition were observed. To determine the impact of cayenne pepper on the gut microbiota or intestinal inflammation, a higher dose of cayenne pepper may be required or participants with inflammatory bowel diseases may need to be studied.

## Figures and Tables

**Table 1:** Population characteristics.

Characteristic	All	BMI < 25	BMI ≥ 25
N	29	9	20
Age, years; mean ± SD	29.5 ± 9.9	25.4 ± 7.6	31.4 ± 10.4
BMI *, kg/m^2^; mean ± SD	29.1 ± 8.20	20.8 ± 1.5	32.9 ± 7.1
Race; *n* (%)			
Asian	11 (37.9%)	5 (55.6%)	6 (30%)
Black	5 (17.2%)	2 (22.2%)	3 (15%)
White	12 (41.4%)	2 (22.2%)	10 (50%)
Other	1 (3.4%)	0 (0%)	1 (5%)
Sex; *n* (%)			
Female	20 (69.0%)	7 (77.8%)	13 (65%)
Male	9 (31.0%)	2 (22.2%)	7 (35%)

* BMI: body mass index.

**Table 2:** Alpha diversity of the gastrointestinal bacterial communities by BMI.

	Alpha Diversity	With Pepper	Without Pepper	*p*-Value
All (*n* = 29)	Chao1	91.9 ± 45.2	88.1 ± 31.2	0.83
	Shannon	2.4 ± 0.3	2.5 ± 0.3	0.70
	Inverse Simpson	6.1 ± 2.4	6.1 ± 2.5	0.73
BMI * < 25 (*n* = 9)	Chao1	87.7 ± 20.5	83 ± 24.4	0.66
	Shannon	2.5 ± 0.4	2.4 ± 0.3	0.17
	Inverse Simpson	6.9 ± 2.8	5.4 ± 1.9	0.14
BMI * ≥ 25 (*n* = 20)	Chao1	93.8 ± 53.1	90.4 ± 34.2	0.99
	Shannon	2.4 ± 0.3	2.5 ± 0.3	0.13
	Inverse Simpson	5.7 ± 2.2	6.3 ± 2.7	0.55

Data are presented as mean ± SD. *p*-value < 0.05 was considered significant. * BMI: body mass index.

**Table 3:** Correlation of gut microbiota taxa at genus level and lipocalin-2 or calprotectin levels.

	Taxa	Coefficient	*p*-Value	*q*-Value
Lipocalin-2	*Blautia*	0.15	0.003	0.10
	*Anaerostipes*	0.20	0.003	0.10
	*Alphaproteobacteria*_unclassified	0.24	0.001	0.10
	*Anaerosporobacter*	0.05	0.009	0.24
Calprotectin	*Eubacterium*	0.15	0.001	0.10
	*Pseudoflavonifractor*	0.12	0.003	0.18

## Data Availability

The data presented in this study are available on request from the corresponding author. The data are not publicly available due to the small sample size.

## Supplementary Materials

The following supporting information can be downloaded at: https://www.mdpi.com/article/10.3390/life12111849/s1, Figure S1: Bar charts of gut bacterial communities for each participant. Figure S2: Stool lipocalin-2 concentrations and beta diversity of gut microbiota. Figure S3: Stool calprotectin concentrations and beta diversity of gut microbiota. Table S1: Alpha diversity of the gastrointestinal bacterial communities by clustering those with the similar responsive gut microbiota to cayenne pepper.
