# Supplementary material for: Minimal Influence of Cayenne Pepper on the Human Gastrointestinal Microbiota and Intestinal Inflammation in Healthy Adult Humans—A Pilot Study
Source: Life (Basel). 2022 Nov 11;12(11):1849. doi: 10.3390/life12111849 (PMC9695709; doi:10.3390/life12111849)
Supplement: Supplementary file 1 [file life-12-01849-s001.zip › life-1986558-supplementary.pdf]

Supplementary Figure S1. Bar charts of gut bacterial communities for each participant. A. All participants. B. Participants with BMI < 25. C. Participants with BMI > 25.

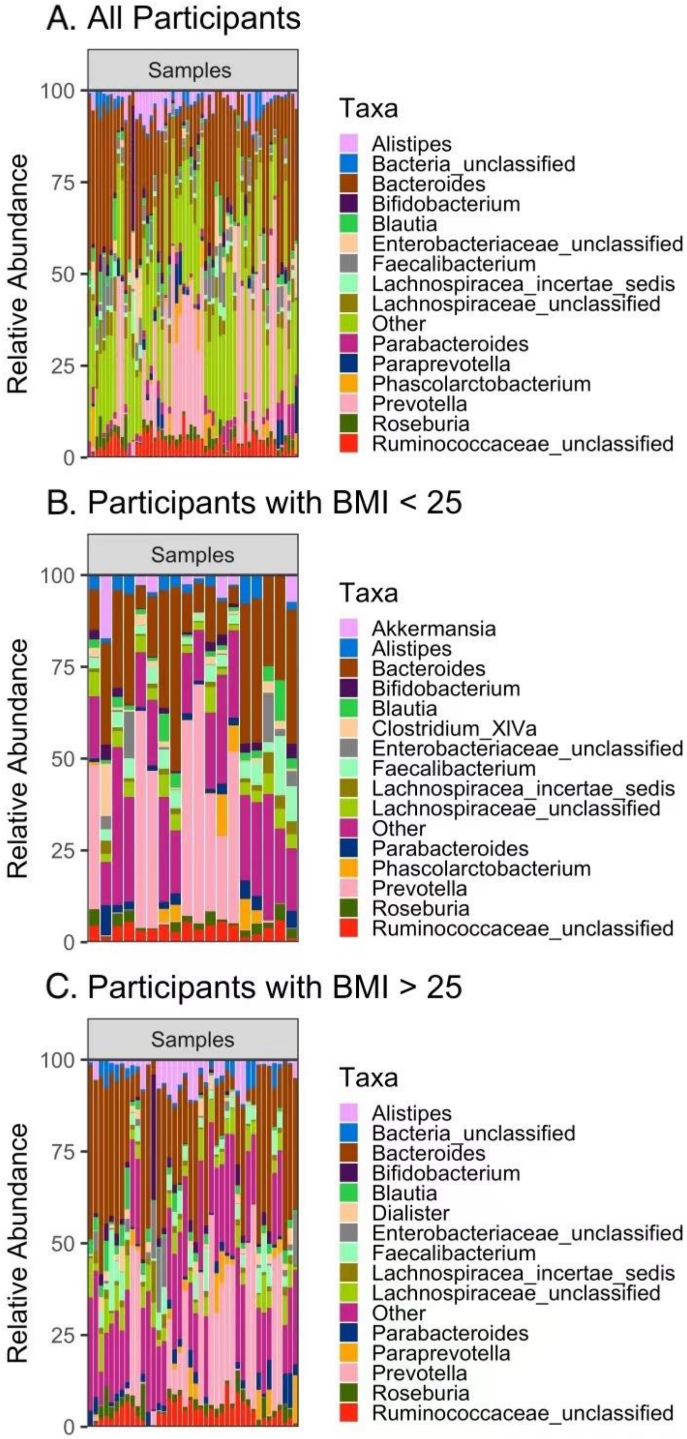

Supplementary Figure S2. Stool lipocalin concentrations were not associated with intestinal bacterial community membership (A, p=0.49; B, p=0.75) or composition (C, p=0.26; D, p=0.39). The gradient on the right of each plot indicates the lipocalin concentration in ng/mL. The darker the circle the higher the lipocalin concentration.

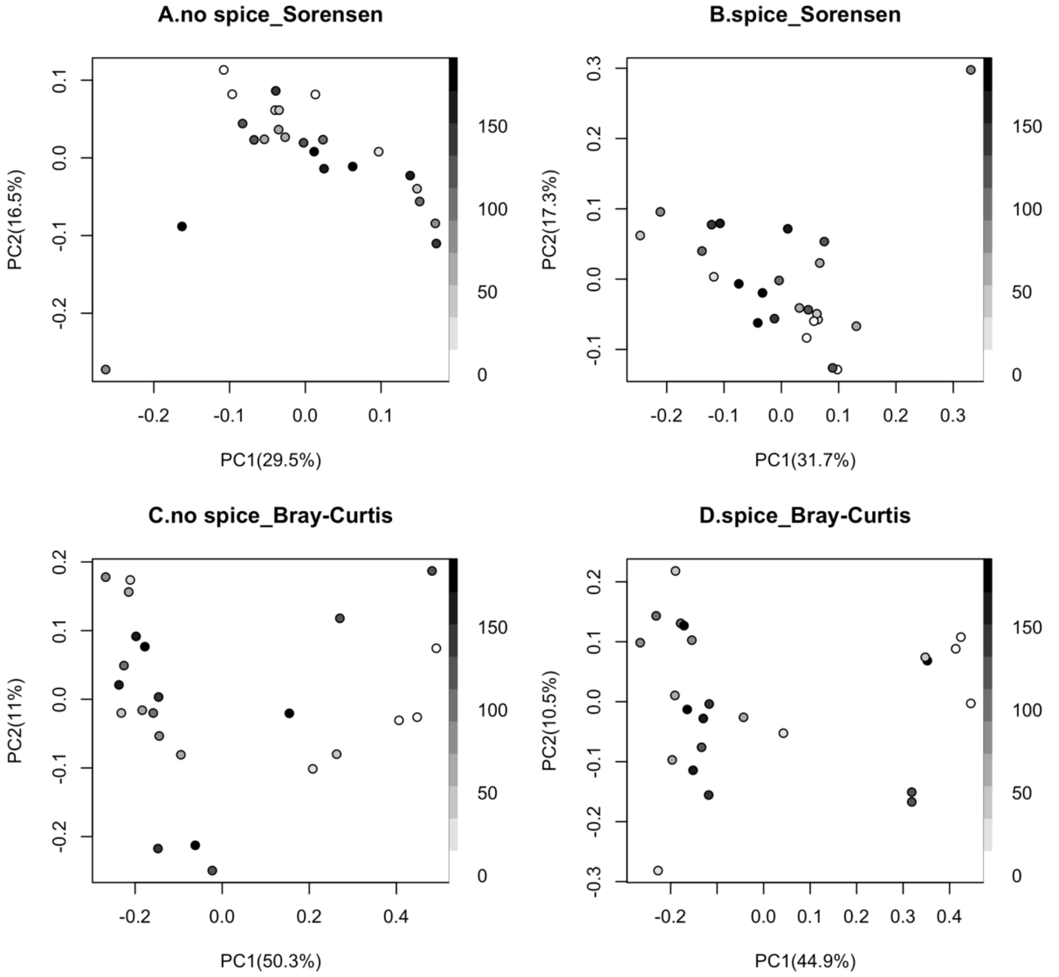

Supplementary Figure S3. Stool calprotectin concentrations were not associated with intestinal bacterial community membership (A, p=0.42; B, p=0.84) or composition (C, p=0.72; D, p=0.79). The gradient on the right of each plot indicates the calprotectin concentration in ng/mL. The darker the circle the higher the calprotectin concentration.

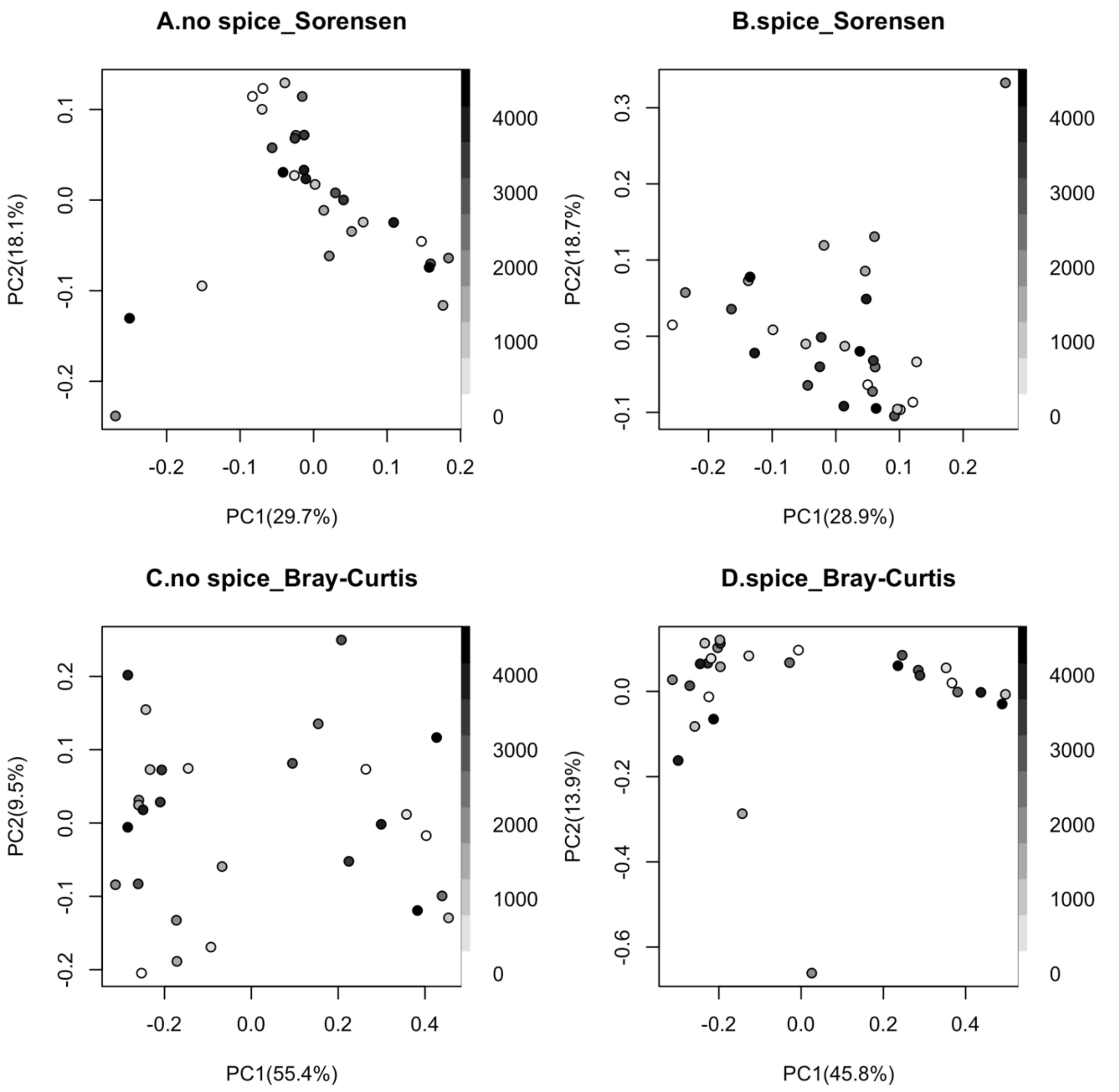

Supplementary Table S1. Alpha diversity of the gastrointestinal bacterial communities by clustering those with similarly responsive gut microbiota to cayenne pepper.

		With spice	Without spice	p-value
Set1 (n=9)	Chao1	108.3 ± 64	100 ± 36.6	0.91
	Shannon	2.5 ± 0.4	2.5 ± 0.4	0.91
	Inverse Simpson	6.2 ± 2.8	6.5 ± 3.6	1.0
Set2 (n=7)	Chao1	80.7 ± 19	92.7 ± 44.8	0. 81
	Shannon	2.3 ± 0.3	2.5 ± 0.2	0.33
	Inverse Simpson	5.2 ± 2.1	5.8 ± 1.5	0.51

Data presented as mean±SD.
